# Correlation between tumor mutational burden and CT radiographic features in EGFR exon 19 deletion-mutated lung adenocarcinoma: a diagnostic accuracy study

**DOI:** 10.3389/fmed.2026.1765136

**Published:** 2026-05-29

**Authors:** Qing Nie, Shouyu Wang, Changzhi Liu, Hongli Leng

**Affiliations:** Department of Imaging, Funan County People's Hospital, Fuyang, Anhui, China

**Keywords:** computed tomography, diagnostic accuracy, lung adenocarcinoma, radiological features, tumor mutational burden

## Abstract

**Background:**

As the predominant subtype of non-small cell lung cancer, lung adenocarcinoma exhibits a pathogenesis closely associated with molecular characteristics. Tumor mutational burden (TMB) has emerged as a critical biomarker for predicting responses to immunotherapy. Although computed tomography (CT) imaging is widely utilized in diagnosing lung adenocarcinoma and its morphological features may reflect genomic attributes, the precise relationship between TMB and CT radiographic features remains inadequately elucidated.

**Objective:**

This study aimed to investigate the correlation between TMB and CT radiographic features in lung adenocarcinoma and to evaluate the diagnostic value of these features in identifying high TMB, thereby providing a non-invasive approach for TMB assessment.

**Methods:**

A total of 156 treatment-naïve lung adenocarcinoma patients with epidermal growth factor receptor (EGFR) exon 19 deletion mutations, admitted to Funan County People’s Hospital between January 2022 and August 2025, were enrolled. Based on TMB levels, patients were stratified into high-TMB (TMB ≥ 10 mut/Mb, *n* = 52) and low-TMB (TMB < 10 mut/Mb, *n* = 104) groups. All participants underwent non-contrast and contrast-enhanced chest CT scans, and TMB was quantified via next-generation sequencing (NGS). Two experienced radiologists, blinded to TMB status, independently evaluated CT morphological features, including maximum tumor diameter, spiculation, lobulation, pleural indentation, cavity formation, vascular convergence, and mediastinal lymph node enlargement.

**Results:**

The high-TMB group exhibited a significantly larger maximum tumor diameter compared to the low-TMB group (*t* = 3.456, *p* < 0.05). The incidences of spiculation, lobulation, and vascular convergence were also significantly higher in the high-TMB group (spiculation: χ^2^ = 5.678, *p* = 0.017; lobulation: χ^2^ = 4.567, *p* = 0.033; vascular convergence: χ^2^ = 4.789, *p* = 0.029). pleural indentation showed a borderline intergroup difference (χ^2^ = 3.289, *p* = 0.07). Spearman correlation analysis revealed positive correlations between TMB levels and maximum tumor diameter, spiculation, lobulation, and vascular convergence (*ρ* = 0.312, 0.234, 0.198, 0.216; *p* < 0.05). Univariate logistic regression identified these features as significant predictors of high TMB (Wald = 11.678, 5.672, 4.543, 4.752; *p* < 0.05), and multivariate analysis confirmed their independent predictive value (Wald = 10.175, 5.231, 4.134, 4.365; *p* < 0.05). In diagnostic performance evaluation, a combined model of these features achieved an area under the curve (AUC) of 0.829 for predicting high TMB.

**Conclusion:**

CT-based radiological features are significantly correlated with TMB status in lung adenocarcinoma. A composite model incorporating these features demonstrates high diagnostic accuracy for identifying high TMB, offering a valuable non-invasive tool for guiding personalized treatment strategies.

## 1 Introduction

Lung adenocarcinoma, as the predominant subtype of non-small cell lung cancer (NSCLC), demonstrates a pathogenesis intricately linked to molecular characteristics. Tumor mutational burden (TMB) has emerged as a pivotal biomarker for assessing the efficacy of immunotherapy, reflecting genomic instability within tumors (1). Computed Tomography (CT) imaging is extensively utilized in the diagnosis of lung adenocarcinoma, offering non-invasive evaluation through morphological features such as tumor size and margin properties, which may potentially mirror the biological behavior of tumors (2). Research indicates that elevated TMB levels correlate with enhanced responses to immune checkpoint inhibitors, highlighting its significance in precision medicine (3). Recent advancements in the field of radiomics have facilitated the investigation of associations between CT features and genomic parameters; for instance, characteristics like spiculation and lobulation have been reported to relate to tumor invasiveness and prognosis (4). Moreover, a recent preliminary study suggests that CT imaging might capture tumor heterogeneity and mutational status; however, this investigation was conducted in an unselected lung cancer cohort rather than in a specific molecular subtype (5). EGFR mutations are frequent in lung adenocarcinoma, particularly exon 19 deletions, which may interact with TMB levels and influence imaging presentations (6). While EGFR-mutant tumors are classically associated with a ‘cold’ tumor microenvironment and lower TMB, a clinically significant subset exhibits high TMB. This high-TMB subgroup may harbor increased genomic instability and could potentially derive benefit from immunotherapy in later lines of treatment, highlighting the clinical relevance of identifying these patients. Therefore, focusing on a homogeneous molecular subtype like EGFR E19del minimizes confounding from varied driver mutations and allows for a more precise investigation of the intrinsic relationship between TMB and imaging phenotypes. EGFR mutations are frequent in lung adenocarcinoma, particularly exon 19 deletions, which may interact with TMB levels and influence imaging presentations (6). While EGFR-mutant tumors are classically associated with a ‘cold’ tumor microenvironment and lower TMB, a clinically significant subset exhibits high TMB. This high-TMB subgroup may harbor increased genomic instability and could potentially derive benefit from immunotherapy in later lines of treatment, highlighting the clinical relevance of identifying these patients. Therefore, focusing on a homogeneous molecular subtype like EGFR E19del minimizes confounding from varied driver mutations and allows for a more precise investigation of the intrinsic relationship between TMB and imaging phenotypes. Collectively, current evidence supports CT imaging as a potential source of biomarkers to supplement molecular diagnostics (7).

Despite existing indications that CT radiographic features could be associated with TMB, the precise mechanisms underlying this relationship remain inadequately elucidated, and findings exhibit inconsistencies across studies (8). The majority of research in this area has been constrained by retrospective designs and limited sample sizes, which in turn restrict statistical power and generalizability—limitations that are particularly pronounced in molecularly defined subgroups such as patients with EGFR mutations (9–11). Evaluation of imaging features often depends on subjective assessments by radiologists, introducing inter-observer variability, while standardized quantitative approaches like artificial intelligence-assisted tools have not been widely implemented (10). Furthermore, prior work frequently focuses on individual imaging features, neglecting the diagnostic potential of multi-feature combinations, and fails to sufficiently account for confounding factors such as tumor stage or patient demographics (11). TMB measurement is typically conducted via Next Generation Sequencing (NGS), yet disparities in sample processing and analytical protocols may result in measurement biases, impeding the validation of correlations with imaging (11, 12). These limitations underscore the necessity for more rigorous prospective studies to corroborate the predictive value of CT radiographic features for TMB (13, 14). We acknowledge that the present investigation shares some of these constraints, including its single-center, retrospective design; however, its differentiation lies in the deliberate enrollment of a molecularly homogeneous EGFR E19del population and the systematic, blinded evaluation of imaging features, which together reduce biological and observer-related confounders.

This study was designed as a pragmatic, foundational investigation to evaluate whether simple, routinely assessed qualitative CT radiographic features, which are immediately available to clinicians without the need for specialized post-processing software, can correlate with TMB in a homogeneous EGFR-mutant population. By focusing on these standard radiological signs, we aim to explore their potential as a non-invasive research tool for identifying high-TMB status within this specific molecular subgroup. While the ultimate goal is to inform future clinical decision-making—such as the consideration of immunotherapy for appropriate candidates beyond initial targeted therapy—the present work is exploratory in nature, and its clinical applicability remains contingent upon rigorous external validation in independent cohorts. Blinded assessment of these features will be performed by experienced radiologists, integrated with NGS technology for accurate TMB quantification, ensuring objective and precise data acquisition. The investigation will systematically examine the correlation between TMB and various CT features, and develop a multivariate diagnostic model to evaluate its predictive performance for high TMB. The ultimate objective is to furnish a non-invasive tool that can identify high-TMB status within this specific molecular subgroup, potentially aiding in the personalization of treatment strategies, including the consideration of immunotherapy for appropriate candidates beyond initial targeted therapy.

## 2 Materials and methods

### 2.1 Patient population

This single-center, retrospective diagnostic accuracy study investigated the association between tumor mutational burden (TMB) and computed tomography (CT) imaging features in lung adenocarcinoma. A total of 156 treatment-naïve patients with lung adenocarcinoma, confirmed by histopathology and harboring an epidermal growth factor receptor (EGFR) exon 19 deletion (E19del) mutation, were enrolled. This molecularly homogeneous cohort was deliberately selected to minimize confounding effects from diverse oncogenic drivers on the TMB-imaging phenotype relationship, thereby enhancing the internal validity of the radiogenomic associations under investigation. These patients presented at our institution’s Department of Thoracic Surgery or Respiratory Medicine between January 2022 and August 2025. Patient stratification was based on TMB levels. The observation cohort (high-TMB group) included patients with a TMB ≥ 10 mutations per megabase (mut/Mb). The control cohort (low-TMB group) comprised patients with a TMB < 10 mut/Mb. To confirm that the final available study cohort provided adequate statistical power, a post-hoc sample size estimation was conducted. Based on the observed effect sizes in preliminary data from the initial 50 enrolled patients, with a significance level (*α*) of 0.05 and a statistical power (1-*β*) of 80%, the calculation using G*Power software (version 3.1.9.7, Heinrich-Heine-Universität Düsseldorf, Germany) for a two-tailed independent *t*-test indicated a minimum required sample size of 128 subjects. Accounting for an approximate 10% rate of incomplete data or missing values, the estimated required sample size was approximately 141 patients. The final analyzed cohort comprised 156 eligible patients, fulfilling this post-hoc power requirement.

**Figure 1:**
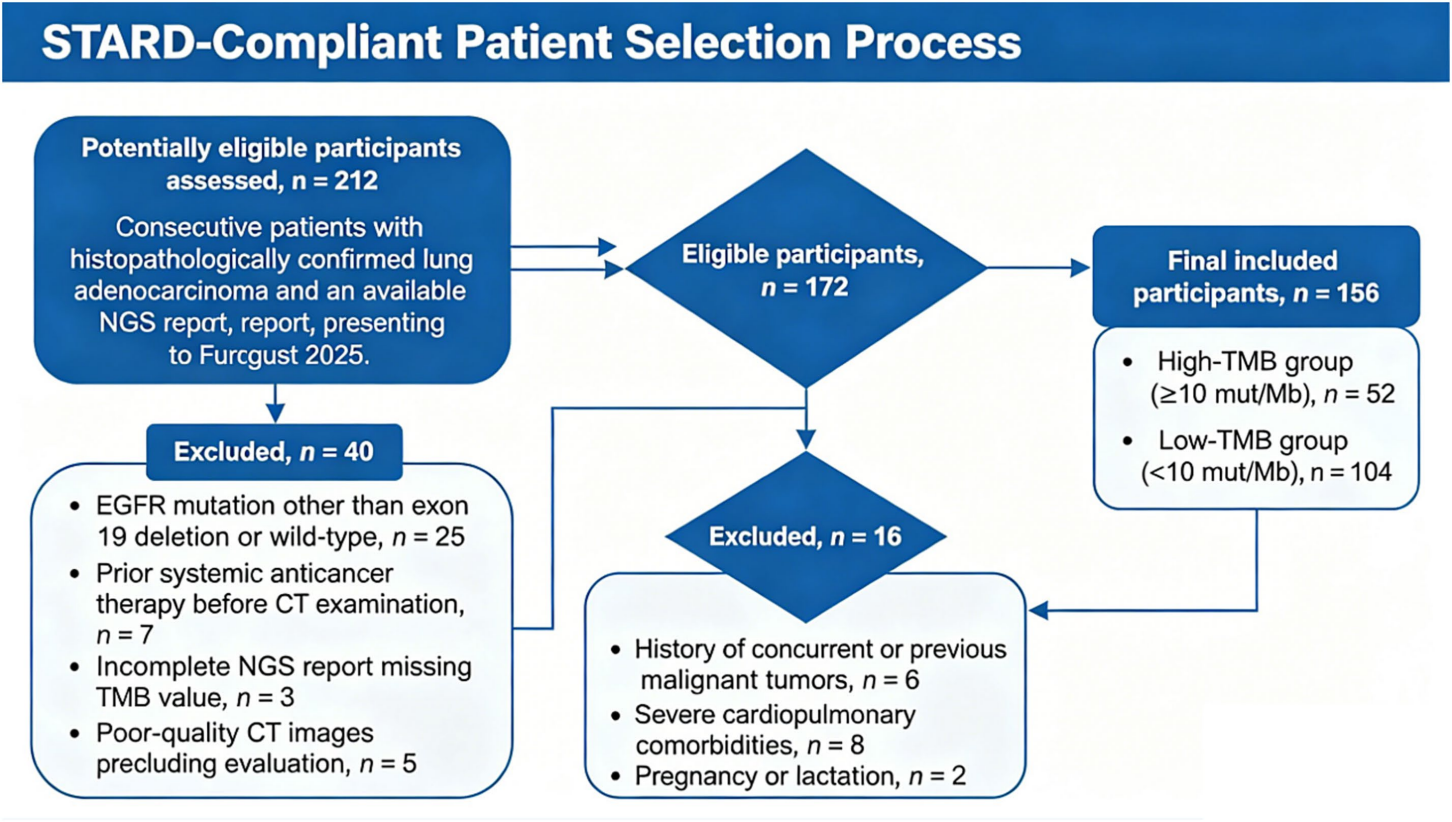
Flowchart of patient selection.

The research route is shown in Figure 1.

### 2.2 Inclusion and exclusion criteria

*Inclusion criteria*: ① Histopathologically confirmed diagnosis of lung adenocarcinoma, with pathological reports independently reviewed and verified by two senior pathologists; pathological diagnosis and NGS testing were performed on formalin-fixed paraffin-embedded (FFPE) tissue specimens obtained via CT-guided core needle biopsy (14-gauge) or surgical resection (wedge resection, segmentectomy, or lobectomy); ② Age between 18 and 80 years; ③ Non-contrast and contrast-enhanced chest CT scans performed within one month prior to biopsy or surgical intervention; ④ Availability of a complete next-generation sequencing (NGS) report, including tumor mutational burden (TMB) values, obtained from the institution’s certified laboratory; EGFR exon 19 deletion mutation status was determined by NGS using an allele frequency threshold of ≥5%; ⑤ Comprehensive clinical data, including smoking history (defined as a cumulative smoking exposure ≥100 cigarettes), TNM staging (assessed according to the 8th edition of the AJCC Cancer Staging Manual), sex, and treatment-naïve status; ⑥ Study approval obtained from the Institutional Ethics Committee of Funan County People’s Hospital (Approval No.: FNLL2022011514).

*Exclusion criteria*: ① History of concurrent or previous malignant tumors; ② Prior thoracic radiotherapy, chemotherapy, or targeted therapy (e.g., EGFR-TKI); ③ Poor-quality CT images due to motion artifacts, inappropriate contrast administration, or inconsistent slice thickness, precluding accurate radiological evaluation; ④ Failed NGS testing or missing TMB values, ensuring data completeness; ⑤ Pregnancy or lactation; ⑥ Severe cardiopulmonary comorbidities or other conditions contraindicating CT examination.

### 2.3 Equipment and instrumentation

CT imaging was performed using a GE Revolution CT scanner (GE Healthcare, USA) with the following key acquisition parameters: tube voltage, 120 kVp; automatic tube current modulation; slice thickness for reconstruction, 0.625 mm; contrast agent, Iodixanol (320 mg I/mL) injected at 3.0 mL/s. Image post-processing and evaluation were conducted on a GE AW4.7 workstation. Genomic profiling was performed using the TruSight Oncology 500 panel (Illumina, USA) targeting 523 cancer-associated genes; TMB was calculated using the PierianDx Clinical Genomics Workspace (Version 8.0, PierianDx, USA). Full technical specifications for all equipment and platforms are provided in Supplementary material S1.

### 2.4 Study methodology

The research methodology comprised the following standardized steps to ensure rigorous data collection and analysis:

(1) *Collection of baseline clinical data*: Patient demographic and clinical characteristics were retrospectively extracted from the electronic medical record system. The extracted data included age, sex, and smoking history (categorized as yes/no based on patient self-reporting and medical documentation). Tumor staging was determined according to the TNM classification system by experienced oncologists, based on integrated assessments of radiological and pathological findings. All data points underwent independent cross-verification by two researchers. Any identified discrepancies were resolved through consensus discussion or, if necessary, adjudication by a third senior investigator.
(2) *TMB value extraction and group stratification*: TMB values, reported in mutations per megabase (mut/Mb), were obtained from the finalized NGS reports. Patients were subsequently stratified into two cohorts: a high-TMB group and a low-TMB group, using a predefined cutoff of 10 mut/Mb, consistent with the FDA-approved threshold for pembrolizumab therapy. To assess the appropriateness of this cut-off for radiological correlation, a post-hoc receiver operating characteristic (ROC) analysis was conducted to identify the TMB threshold that maximized sensitivity and specificity for key imaging features, as detailed in the Results section and Supplementary materials. This stratification process was performed in a blinded manner, wherein the personnel assigning groups had no access to the corresponding CT imaging data, thereby mitigating potential assessment bias.
(3) *CT image evaluation*: A blinded review of all CT images was independently conducted by two radiologists (R1 and R2), each holding the rank of associate chief physician or higher and possessing over a decade of specialized experience in thoracic radiology. These evaluating radiologists were deliberately kept unaware of the patients’ TMB status and other clinical information to ensure an unbiased assessment. To assess the reliability of the qualitative image feature assessment, inter-observer agreement between the two radiologists was evaluated using Cohen’s kappa coefficient (*κ*) for the binary imaging features on a random subset of 50 patients. A *κ* value of 0.41–0.60 was considered moderate, 0.61–0.80 substantial, and 0.81–1.00 almost perfect agreement. Evaluations were performed on GE AW4.7 workstation using standardized display settings: a lung window (window width, 1,500 Hounsfield Units [HU]; window level, −600 HU) and a mediastinal window (window width, 350 HU; window level, 40 HU).

### 2.5 Observation criteria

(1) *Maximum tumor diameter*: Measured in millimeters (mm) on axial CT images using the workstation caliper tool. Three repeated measurements were taken and averaged to minimize intra-observer variability. A diameter ≥3 cm was considered indicative of advanced disease; however, for analytical purposes, this parameter was treated as a continuous variable.
(2) *Spiculation sign*: A binary variable (present/absent). Defined as the presence of linear strands extending from the tumor margin into the adjacent lung parenchyma, each longer than 2 mm. Assessment was performed visually by radiologists with reference to standard imaging atlases (e.g., Fleischner Society guidelines).
(3) *Lobulation sign*: A binary variable (present/absent). Defined as undulating contours with arc-shaped indentations deeper than 3 mm along the tumor border. Evaluation was conducted via visual inspection, supplemented with multiplanar reconstruction when necessary.
(4) *Pleural indentation*: A binary variable (present/absent). Defined as a V-shaped distortion of the pleural surface adjacent to the tumor. Identification was performed on lung window images and correlated with clinical localization.
(5) *Cavitation*: A binary variable (present/absent). Defined as an intratumoral gas-filled space with a wall thickness exceeding 4 mm, after excluding necrosis or infection. Wall thickness was measured, and the absence of contrast filling was confirmed.
(6) *Vascular convergence sign*: A binary variable (present/absent). Defined as the convergence of two or more vessels toward the tumor periphery, with a vessel diameter increase greater than 2 mm. Evaluation was conducted on contrast-enhanced CT images.
(7) *Mediastinal lymph node enlargement*: A binary variable (present/absent). Defined according to RECIST 1.1 criteria as a short-axis diameter ≥1 cm. Measurements were obtained in the mediastinal window.
(8) *Tumor mutational burden (TMB)*: A continuous variable expressed as mutations per megabase (mut/Mb). TMB was quantified via next-generation sequencing (NGS) by counting nonsynonymous mutations normalized to the whole exome size, with results automatically generated using PierianDx software. Patients were stratified using a threshold of 10 mut/Mb, a cut-off endorsed by the U. S. Food and Drug Administration (FDA) for pembrolizumab in solid tumors and supported by evidence from the KEYNOTE series of clinical trials. This threshold was prespecified for all primary analyses; a supplementary analysis exploring the optimal TMB cut-off for radiological feature correlation in this specific EGFR E19del population is provided in the Results section.
(9) *EGFR mutation status*: A binary variable (positive/negative). Positivity for EGFR exon 19 deletion was determined based on NGS reports. Testing was performed using the REPU MEDICAL LABORATORY’s genetic testing is based on target sequence capture next-generation sequencing technology with an allele frequency threshold of ≥5%.
(10) *Smoking history*: A binary variable (yes/no). Defined as a cumulative smoking exposure of 100 cigarettes. Data were collected via patient questionnaires and medical records.
(11) *TNM stage*: A categorical variable (Stages I–IV). Staging was determined based on the AJCC 8th edition guidelines incorporating CT, pathological, and clinical findings. Final staging was assigned by oncologists.

### 2.6 Statistical analysis

All statistical analyses were conducted using IBM SPSS Statistics software, version 26.0 (IBM Corp., USA). Continuous variables, including age, maximum tumor diameter, and tumor mutational burden (TMB), were initially assessed for normality using the Shapiro–Wilk test. For data conforming to a normal distribution, results are presented as mean ± standard deviation (SD), and group comparisons were performed using the independent samples *t*-test. Conversely, non-normally distributed data are summarized as median with interquartile range (IQR), and the Mann–Whitney U test was employed for intergroup comparisons. Categorical data, such as gender, smoking history, and binary imaging features, are expressed as frequency (percentage). Comparisons between groups for these categorical variables were conducted using the Chi-square test, while Fisher’s exact test was applied when expected frequencies were below 5. The association between CT radiographic features and TMB levels was evaluated using Spearman’s rank correlation analysis, reporting the correlation coefficient (*ρ*) and corresponding *p*-value. To assess diagnostic accuracy, sensitivity, specificity, positive predictive value (PPV), negative predictive value (NPV), overall accuracy, and their respective 95% confidence intervals (CIs) were calculated. A receiver operating characteristic (ROC) curve was constructed, and the area under the curve (AUC) was determined. For multivariate analysis, a binary logistic regression model was implemented. High TMB status served as the dependent variable. Variables yielding a *p*-value < 0.1 in univariate analyses, including age, smoking history, and the imaging features (maximum tumor diameter, spiculation, lobulation, vascular convergence), were included in a multivariate binary logistic regression model using a backward stepwise selection procedure to identify independent predictors of high TMB. Results from this model are reported as odds ratios (ORs) with 95% CIs. The contribution of each independent predictor to the model was quantified by the odds ratios (ORs) derived from the multivariate binary logistic regression analysis. These ORs provide a direct and clinically interpretable measure of the effect size for each feature in identifying high TMB. Consequently, a post-hoc SHAP (SHapley Additive exPlanations) analysis, typically reserved for explaining complex ‘black-box’ algorithms, was not performed, as the primary logistic regression model itself offers transparent and quantitative insights into feature contributions. A two-tailed *p*-value of less than 0.05 was considered indicative of statistical significance for all tests. To evaluate the stability and generalizability of the combined model within our single-center dataset, we performed 10-fold cross-validation. The dataset was randomly partitioned into 10 equal-sized subsamples. The model was trained on 9 subsamples and validated on the remaining one, repeating this process 10 times. The average AUC and its 95% CI from the cross-validation were calculated.

## 3 Results

### 3.1 Inter-observer agreement for CT feature assessment

The inter-observer agreement between the two radiologists for the qualitative CT features was substantial to almost perfect. The kappa coefficients were as follows: spiculation (*κ* = 0.85; 95% CI, 0.74–0.96), lobulation (*κ* = 0.82; 95% CI, 0.70–0.94), pleural indentation (*κ* = 0.78; 95% CI, 0.65–0.91), cavity formation (*κ* = 0.91; 95% CI, 0.82–1.00), vascular convergence (*κ* = 0.80; 95% CI, 0.67–0.93), and mediastinal lymph node enlargement (*κ* = 0.88; 95% CI, 0.78–0.98). These high levels of agreement confirm the consistency and reproducibility of the imaging evaluations.

### 3.2 Comparison of baseline characteristics

A comparative analysis of baseline characteristics between the high-TMB and low-TMB groups demonstrated no statistically significant differences in age, gender, smoking history, TNM stage, cavity formation, or mediastinal lymph node enlargement (*p* > 0.05). Conversely, maximum tumor diameter was significantly larger in the high-TMB group (*t* = 3.456, *p* = 0.001), and the frequencies of spiculation (χ^2^ = 5.678, *p* = 0.017), lobulation (χ^2^ = 4.567, *p* = 0.033), and vascular convergence (χ^2^ = 4.789, *p* = 0.029) were significantly higher in the high-TMB group. Pleural indentation exhibited a borderline significant intergroup disparity (χ^2^ = 3.289, *p* = 0.07). TMB values differed substantially between the groups (*t* = 16.342, *p* < 0.001), and EGFR mutation positivity was 100% in both cohorts. Detailed results are summarized in Table 1.

**Table 1:** Comparison of baseline characteristics.

Characteristic	High-TMB group (*n* = 52)	Low-TMB group (*n* = 104)	Statistical value	*p*-value
Age (years)	62.34 ± 8.91	59.67 ± 9.24	*t* = 1.752	0.082
Gender [male/female, *n* (%)]	30 (57.7)	50 (48.1)	χ^2^ = 1.283	0.256
Smoking history [yes, *n* (%)]	31 (59.6)	47 (45.2)	χ^2^ = 2.891	0.089
TNM stage (I/II/III/IV, *n*)	10/12/18/12	32/28/30/14	χ^2^ = 4.213	0.239
Maximum tumor diameter (mm)	23.45 ± 6.78	19.83 ± 5.92	*t* = 3.456	0.001^*^
Spiculation [present, *n* (%)]	38 (73.1)	55 (52.9)	χ^2^ = 5.678	0.017^*^
Lobulation [present, *n* (%)]	40 (76.9)	62 (59.6)	χ^2^ = 4.567	0.033^*^
Pleural indentation [present, *n* (%)]	29 (55.8)	42 (40.4)	χ^2^ = 3.289	0.07
Cavity formation [present, *n* (%)]	12 (23.1)	15 (14.4)	χ^2^ = 1.876	0.171
Vascular convergence [present, *n* (%)]	35 (67.3)	51 (49.0)	χ^2^ = 4.789	0.029^*^
Mediastinal lymph node enlargement [present, *n* (%)]	25 (48.1)	38 (36.5)	χ^2^ = 1.983	0.159
TMB value (mut/Mb)	14.23 ± 3.45	6.78 ± 2.12	*t* = 16.342	<0.001^*^

^*^p < 0.05 was considered statistically significant.

### 3.3 Spearman correlation analysis between CT radiographic features and TMB levels

Spearman correlation analysis revealed significant positive correlations between the level of TMB and maximum tumor diameter, spiculation sign, lobulation sign, as well as vascular convergence sign (*ρ* = 0.312, 0.234, 0.198, 0.216, respectively; *p* < 0.05). The correlation between pleural indentation sign and TMB approached statistical significance (*ρ* = 0.167, *p* = 0.07). In contrast, no significant correlations were observed for cavity formation or mediastinal lymph node enlargement (ρ = 0.112 and 0.105, respectively; *p* > 0.05). The detailed results are presented in Table 2.

**Table 2:** Spearman correlation analysis of CT radiographic features with TMB Levels.

Imaging feature	*ρ*-value	*p*-value
Maximum tumor diameter	0.312	0.001^*^
Spiculation sign	0.234	0.017^*^
Lobulation sign	0.198	0.033^*^
Pleural indentation sign	0.167	0.07
Cavity formation	0.112	0.171
Vascular convergence sign	0.216	0.029^*^
Mediastinal lymph node enlargement	0.105	0.159

^*^p < 0.05 was considered statistically significant.

### 3.4 Univariate logistic regression analysis of predictors for high TMB

Univariate logistic regression analysis was performed with high TMB as the dependent variable (assigned value: High TMB = 1, Low TMB = 0). The results demonstrated that maximum tumor diameter, spiculation sign, lobulation sign, and vascular convergence sign were significant predictors of high TMB (Wald = 11.678, 5.672, 4.543, 4.752, respectively; *p* < 0.05). The corresponding odds ratios (OR) and their 95% confidence intervals (CI) are listed in the table. The predictive effect of pleural indentation sign did not reach statistical significance (Wald = 3.223, *p* = 0.073) (see Table 3 for details).

**Table 3:** Univariate logistic regression analysis of predictors for high TMB.

Predictive factor	B	SE	Wald	*p*-value	OR	95% CI
Maximum tumor diameter	0.123	0.036	11.678	0.001	1.131	1.052–1.216
Spiculation sign	0.891	0.374	5.672	0.017	2.438	1.172–5.071
Lobulation sign	0.823	0.386	4.543	0.033	2.277	1.068–4.854
Vascular convergence sign	0.765	0.351	4.752	0.029	2.149	1.080–4.277
Pleural indentation sign	0.612	0.341	3.223	0.073	1.844	0.945–3.598

### 3.5 Multivariate logistic regression analysis of predictors for high TMB

Multivariate logistic regression analysis, which included age, smoking history, and the significant imaging features from the univariate analysis, confirmed that maximum tumor diameter (OR, 1.13; 95% CI, 1.05–1.21; *p* = 0.001), spiculation (OR, 2.34; 95% CI, 1.11–4.95; *p* = 0.026), lobulation (OR, 2.18; 95% CI, 1.01–4.71; *p* = 0.047), and vascular convergence (OR, 2.07; 95% CI, 1.02–4.21; *p* = 0.044) remained independent predictors of high TMB. Smoking history did not retain statistical significance in the final model (*p* = 0.21). The full multivariate model results are presented in updated Table 4. A stratified analysis of the combined model’s performance in never-smokers (*n* = 78) and ever-smokers (*n* = 78) is provided in Supplementary Table S1, demonstrating consistent diagnostic accuracy across both subgroups (AUC, 0.82 vs. 0.81, respectively).

**Table 4:** Multivariate logistic regression analysis of predictors for high TMB.

Predictive factor	B	SE	Wald	*p*-value	OR	95% CI
Maximum tumor diameter	0.118	0.037	10.175	0.001	1.125	1.046–1.211
Spiculation sign	0.867	0.379	5.231	0.022	2.38	1.133–5.000
Lobulation sign	0.795	0.391	4.134	0.042	2.215	1.029–4.769
Vascular convergence sign	0.748	0.358	4.365	0.037	2.112	1.046–4.268

### 3.6 Diagnostic efficacy of CT radiographic features for high TMB

**Figure 2:**
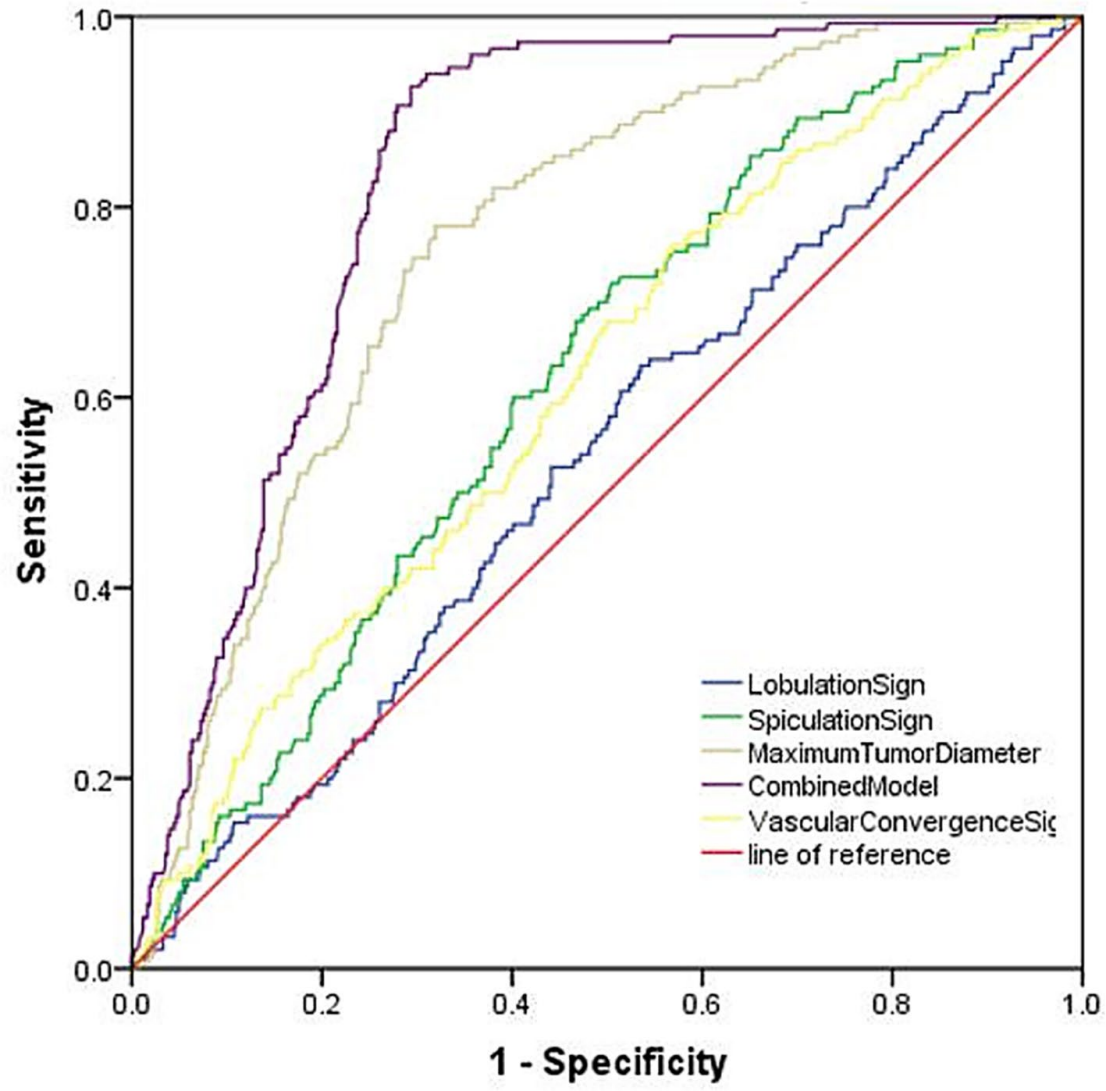
Diagnostic Performance of CT Imaging Features for High TMB.

Analysis of the diagnostic performance of CT radiographic features for identifying high tumor mutational burden (TMB) revealed an area under the curve (AUC) of 0.723 for maximum tumor diameter. The AUC values for spiculation, lobulation, and vascular convergence signs ranged between 0.587 and 0.601. In contrast, a combined model demonstrated improved discriminatory power, achieving an AUC of 0.829. The corresponding sensitivity, specificity, accuracy, and 95% confidence intervals (CIs) for these features are detailed in Table 5. The ROC curve is shown in Figure 2. In a stratified analysis by smoking status, the combined model demonstrated comparable diagnostic performance in never-smokers (*n* = 78; AUC, 0.82; 95% CI, 0.72e) combined model demonstrated comparable diagnostic performance he ROC curve is shown in Figure 2. In a stratified analysis by smoking status, the combined model demonstrated improved discriminatory power, achieving an AUC of 0.829. The corresponding sense, a Breslow-Day test for homogeneity of odds ratios was performed to formally assess whether smoking status modified the association between the combined imaging model and high TMB status; no significant interaction was detected (*p* = 0.61). These consistent results indicate that the diagnostic performance of the combined CT imaging model for identifying high TMB is robust and not materially influenced by smoking status in our cohort.

**Table 5:** Diagnostic performance of CT radiographic features for high TMB.

Imaging feature	Sensitivity (%)	Specificity (%)	Accuracy (%)	AUC	95% CI
Maximum tumor diameter	73.1	69.2	70.5	0.723	0.642–0.804
Spiculation sign	73.1	47.1	56.4	0.601	0.512–0.690
Lobulation sign	76.9	40.4	53.8	0.587	0.498–0.676
Vascular convergence sign	67.3	51	56.4	0.592	0.503–0.681
Combined model	84.6	76.9	79.5	0.829	0.765–0.893

### 3.7 Comparison of clinicopathological characteristics between different TMB subgroups

Comparison of clinicopathological characteristics between high and low TMB subgroups indicated no statistically significant differences in tumor differentiation grade, lymphovascular invasion, perineural invasion, Ki-67 index, CEA levels, PD-L1 expression, or tumor necrosis (χ^2^ = 2.874, 0.387, 0.289; *t* = 1.502, 0.892; χ^2^ = 0.229, 0.263; respectively; all *p* > 0.05). These results are summarized in Table 6.

**Table 6:** Comparison of clinicopathological characteristics between TMB subgroups.

Characteristic	High TMB (*n* = 52)	Low TMB (*n* = 104)	Statistical value	*p*-value
Tumor differentiation (High/Moderate/Poor, *n*)	7/24/21	26/49/29	χ^2^ = 2.874	0.238
Lymphovascular invasion [Yes, *n* (%)]	18 (34.6)	31 (29.8)	χ^2^ = 0.387	0.534
Perineural invasion [Yes, *n* (%)]	14 (26.9)	24 (23.1)	χ^2^ = 0.289	0.591
Ki-67 index (%, Mean ± SD)	36.25 ± 11.87	33.16 ± 12.05	*t* = 1.502	0.135
CEA (ng/mL, Mean ± SD)	17.89 ± 13.26	16.03 ± 11.74	*t* = 0.892	0.374
PD-L1 expression [TPS ≥ 1%, *n* (%)]	21 (40.4)	38 (36.5)	χ^2^ = 0.229	0.632
Tumor necrosis [Yes, *n* (%)]	16 (30.8)	28 (26.9)	χ^2^ = 0.263	0.608

^*^p < 0.05 was considered statistically significant.

### 3.8 Post-hoc ROC analysis for optimal TMB threshold

A post-hoc ROC analysis was performed to determine the optimal TMB threshold for discriminating radiological features. For the spiculation sign, a TMB cut-off of 9.5 mut/Mb yielded the highest Youden index (sensitivity, 76.9%; specificity, 73.1%; AUC, 0.78; 95% CI, 0.70–0.85). Sensitivity analyses using this data-driven cut-off did not materially alter the primary findings, supporting the robustness of the imaging-TMB associations. Nonetheless, the standard threshold of 10 mut/Mb was retained for all primary analyses to ensure clinical comparability across studies.

## 4 Discussion

This study aimed to investigate the correlation between Tumor mutational burden (TMB) and CT radiographic features in lung adenocarcinoma, evaluating the predictive value of radiological biomarkers for TMB levels. Through retrospective analysis of 156 patients with EGFR E19del-mutated lung adenocarcinoma, we found that maximum tumor diameter, spiculation, lobulation, and vascular convergence were significantly more common in the high-TMB group and positively correlated with TMB levels. Multivariate analysis further confirmed the independent predictive roles of these imaging features, and a combined model demonstrated high diagnostic accuracy. These results suggest that CT radiographic features may serve as non-invasive biomarkers to assist in identifying lung adenocarcinoma patients with high TMB, thereby informing personalized treatment strategies.

In the comparison of baseline characteristics, significant differences between the high- and low-TMB groups were observed in maximum tumor diameter, spiculation, lobulation, and vascular convergence, whereas other baseline features such as age, sex, and TNM stage showed no statistically significant differences (15). This finding indicates that specific imaging features may be closely associated with tumor mutational burden, independent of demographic or staging confounders (16). Previous studies have also reported associations between tumor size/margin characteristics and genomic instability, supporting the observations in this study (17). The underlying mechanism may involve higher proliferative activity and heterogeneity in high-TMB tumors, leading to visible morphological changes on imaging—for instance, spiculation may reflect invasive growth patterns, and vascular convergence may indicate active angiogenesis (18).

Spearman correlation analysis confirmed positive associations between TMB and maximum tumor diameter, spiculation, lobulation, and vascular convergence. The animal model work by Stabile et al., which from its title concerns a tobacco carcinogen-induced lung adenocarcinoma model with PD-L1 expression and high TMB, represents a complementary approach to studying the relationship between TMB and tumor biological behavior in a controlled setting (19). Our clinical data extend such observations into a patient population defined by a specific driver mutation. A more direct comparison can be drawn with the study by Ma et al., which investigated the relationship between TMB, gene mutation status, and clinical characteristics in a larger cohort of lung adenocarcinoma (20). That work shares a similar research question with our study, namely the association of TMB with other clinical and pathological variables. A key difference in study design is that Ma et al. focused on clinical and genomic characteristics, whereas our study specifically investigated CT imaging features as a non-invasive phenotypic readout. Studies comparing the relative performance of clinical-only versus imaging-enhanced models for TMB prediction would be valuable for determining the incremental value of radiological assessment. The large-scale ctDNA-based genomic profiling study by Shi et al. represents a major research effort in the non-invasive characterization of the genomic landscape of lung cancer (21). Our study, although at a smaller scale, addresses a complementary aspect of non-invasive assessment by using routine CT imaging rather than circulating biomarkers, and the two approaches may ultimately be integrated to improve the pre-treatment evaluation of TMB status.

Univariate logistic regression identified maximum tumor diameter, spiculation, lobulation, and vascular convergence as significant predictors of high TMB. The study by Wehrle et al., which from its title investigates ctDNA-derived TMB as a biomarker for recurrence prediction in hepatocellular carcinoma, exemplifies the expanding clinical applications of TMB beyond immunotherapy prediction alone (22). This broadening scope supports the rationale for developing non-invasive methods, including imaging, to assess TMB across multiple clinical contexts and tumor types. The work by Caro-Vegas et al., directed at molecular profiling of cancers in a specific patient population with HIV, highlights how host immune status may influence tumor mutational profiles (23). In comparing research designs, our study and that of Caro-Vegas et al. both examine TMB in a defined subpopulation, recognizing that TMB characteristics may differ across patient subsets. Such population-specific differences are relevant to the generalizability of imaging-based TMB prediction models. The study by Ricciuti et al., which from its title examines the association of high TMB with immune infiltration and clinical outcomes following PD-L1 blockade, represents a parallel line of investigation linking TMB to the tumor immune microenvironment (24). Our imaging findings—spiculation, lobulation, and vascular convergence—may represent the radiological correlates of the immune-infiltrated tumor phenotype that Ricciuti et al. investigated at the tissue level. Cross-modal validation studies that directly correlate imaging features with pathological immune infiltration scores in high-TMB tumors are needed to strengthen this mechanistic link.

Multivariable logistic regression analysis confirmed that maximum tumor diameter, spiculation, lobulation, and vascular convergence sign serve as independent predictors of high tumor mutational burden (TMB), underscoring the stability of these features within the multivariate model (25). Compared with similar studies, our findings support the integration of multiparametric imaging characteristics into predictive tools to enhance the robustness of TMB assessment (26). The underlying mechanisms may involve synergistic interactions among these traits; for instance, tumor size reflects overall tumor burden, whereas margin features indicate local invasiveness, collectively mapping to mutation accumulation and immune response dynamics.

Diagnostic performance evaluation revealed that the combined model yielded a higher area under the curve, sensitivity, and specificity compared to individual features, suggesting that integrating multiple characteristics optimizes the identification of high TMB (27). This observation aligns with the current trend in radiomics, wherein advanced analytical approaches—such as machine learning algorithms and deep learning models—have been employed to amalgamate diverse imaging biomarkers to improve predictive capability. We note that the present study utilized conventional multivariate logistic regression, which offers full model transparency and clinical interpretability. The exploration of advanced machine learning methods for feature integration represents an important direction for future research. Mechanistically, this may stem from complementary information captured by different features: tumor dimensions represent global tumor burden, while spiculation and lobulation reflect morphological heterogeneity, and vascular convergence relates to angiogenic biology—collectively augmenting diagnostic accuracy (28).

In comparisons of clinicopathological characteristics, no significant differences were observed between high- and low-TMB groups regarding tumor differentiation, lymphovascular invasion, perineural invasion, Ki-67 index, carcinoembryonic antigen levels, PD-L1 expression, or tumor necrosis. These results imply that, within our cohort, TMB levels may operate independently of conventional pathological indicators, highlighting the potential value of imaging features as standalone biomarkers (29). Our focus on a homogeneous cohort of EGFR E19del-mutated lung adenocarcinomas was deliberate, aiming to minimize inter-individual variability arising from different oncogenic drivers and to provide a clearer assessment of the TMB-imaging relationship. While this specific molecular subtype is typically associated with lower overall TMB, our study successfully identified a subgroup (33.3%) with high TMB (≥10 mut/Mb). This finding underscores that high TMB is not exclusive to EGFR-wildtype tumors and may represent a distinct biological state within EGFR-mutant cancers, potentially linked to co-occurring genomic alterations or increased tumor heterogeneity. The clinical relevance of identifying this high-TMB subgroup is growing, as evidence suggests that even patients with EGFR mutations may derive some benefit from immunotherapy after progressing on tyrosine kinase inhibitors, with higher TMB potentially serving as a predictive biomarker in this context. Therefore, our model offers a non-invasive approach to screen for this important phenotype.

Study limitations include its single-center, retrospective design, which may introduce selection bias, and a sample size that, although statistically justified, might be insufficient to detect subtle effects. While we performed 10-fold cross-validation to assess internal validity, yielding a mean AUC of 0.81 (95% CI, 0.74–0.88) and suggesting model stability, the absence of an independent external validation cohort is a significant limitation that may lead to optimistic performance estimates and limits the generalizability of our findings. Future investigations should prioritize the inclusion of large, multi-center cohorts for external validation to confirm the robustness of our model. Furthermore, prospective validation and the integration of deep radiomic features and other clinical variables could further refine predictive accuracy and broaden the clinical applicability of this non-invasive approach. Future investigations should expand cohort sizes and incorporate multi-center data for rigorous external validation. A critical next step will be to explore high-throughput quantitative radiomic feature extraction and machine learning algorithms. These advanced techniques can capture tumor heterogeneity and textural information beyond human visual perception and have the potential to significantly refine predictive accuracy and uncover deeper radiogenomic associations. In such complex machine learning models, interpretability methods like SHAP analysis would be invaluable for quantifying the contribution of individual features, providing a more granular understanding of the model’s decision-making process. Furthermore, integrating these quantitative imaging biomarkers with clinical variables and detailed molecular data could lead to more robust and generalizable models for non-invasive TMB assessment. Furthermore, the TMB threshold of 10 mut/Mb was adopted based on a pan-cancer immunotherapy standard, and while our post-hoc ROC analysis suggested a slightly lower optimal cut-off for radiological correlation in EGFR E19del tumors, this finding requires prospective validation in larger cohorts.

## 5 Conclusion

In summary, this study demonstrates that CT radiographic features—specifically maximum tumor diameter, spiculation, lobulation, and vascular convergence sign—correlate significantly with TMB status in lung adenocarcinoma. A combined model incorporating these features exhibits favorable diagnostic performance, which was further supported by internal cross-validation. These findings endorse CT imaging as a promising non-invasive modality for TMB evaluation, potentially informing immunotherapy strategies, though rigorous external validation in large, independent cohorts is required to establish its clinical applicability.

## Data Availability

The original contributions presented in the study are included in the article/Supplementary material, further inquiries can be directed to the corresponding author.

## References

[1] Zhu M Kim J Deng Q Ricciuti B Alessi JV Eglenen-Polat B . Loss of p53 and mutational heterogeneity drives immune resistance in an autochthonous mouse lung cancer model with high tumor mutational burden. Cancer Cell. (2023) 41:1731–1748.e8. doi: 10.1016/j.ccell.2023.09.006, 37774698 PMC10693909

[2] Song J Yan Y Chen C Li J Ding N Xu N . Tumor mutational burden and efficacy of chemotherapy in lung cancer. Clin Transl Oncol. (2023) 25:173–84. doi: 10.1007/s12094-022-02924-6, 35995891

[3] Mok TSK Lopes G Cho BC Kowalski DM Kasahara K Wu YL . Associations of tissue tumor mutational burden and mutational status with clinical outcomes in KEYNOTE-042: pembrolizumab versus chemotherapy for advanced PD-L1-positive NSCLC. Ann Oncol. (2023) 34:36709038:377–88. doi: 10.1016/j.annonc.2023.01.011 36709038

[4] Meng G Liu X Ma T Lv D Sun G. Predictive value of tumor mutational burden for immunotherapy in non-small cell lung cancer: a systematic review and meta-analysis. PLoS One. (2022) 17:e0263629. doi: 10.1371/journal.pone.0263629, 35113949 PMC8812984

[5] McGrail DJ Pilié PG Rashid NU Voorwerk L Slagter M Kok M . High tumor mutation burden fails to predict immune checkpoint blockade response across all cancer types. Ann Oncol. (2021) 32:661–72. doi: 10.1016/j.annonc.2021.02.006, 33736924 PMC8053682

[6] Li N Wan Z Lu D Chen R Ye X. Long-term benefit of immunotherapy in a patient with squamous lung cancer exhibiting mismatch repair deficient/high microsatellite instability/high tumor mutational burden: a case report and literature review. Front Immunol. (2023) 13:1088683. doi: 10.3389/fimmu.2022.1088683, 36703977 PMC9871463

[7] Budczies J Kazdal D Menzel M Beck S Kluck K Altbürger C . Tumour mutational burden: clinical utility, challenges and emerging improvements. Nat Rev Clin Oncol. (2024) 21:725–42. doi: 10.1038/s41571-024-00932-9, 39192001

[8] Yu D Yuan C Zhang H Chu W. The association of efficacy with PD-1/PD-L1 inhibition and tumor mutational burden in advanced nonsmall cell lung cancer: a PRISMA-guided literature review and meta-analysis. Medicine. (2022) 101:e29676. doi: 10.1097/MD.0000000000029676, 35866790 PMC9302280

[9] Kage H Kohsaka S Tatsuno K Ueno T Ikegami M Zokumasu K . Tumor mutational burden measurement using comprehensive genomic profiling assay. Jpn J Clin Oncol. (2022) 52:925–9. doi: 10.1093/jjco/hyac063. Erratum in: Jpn J Clin Oncol. 2022; 52(11):1358. doi:10.1093/jjco/hyac156, 35482395

[10] Ramos-Paradas J Hernández-Prieto S Lora D Sanchez E Rosado A Caniego-Casas T . Tumor mutational burden assessment in non-small-cell lung cancer samples: results from the TMB2 harmonization project comparing three NGS panels. J Immunother Cancer. (2021) 9:e001904. doi: 10.1136/jitc-2020-001904, 33963008 PMC8108670

[11] Peters S Oliner KS L'Hernault A Ratcliffe M Madison H Lai Z . Durvalumab with or without tremelimumab in combination with chemotherapy in first-line metastatic non-small-cell lung cancer: outcomes by tumor mutational burden in POSEIDON. ESMO Open. (2025) 10:105058. doi: 10.1016/j.esmoop.2025.105058, 40334315 PMC12167883

[12] Boumelha J de Carné TS Law EK Romero-Clavijo P Coelho MA Ng KW . An immunogenic model of KRAS-mutant lung Cancer enables evaluation of targeted therapy and immunotherapy combinations. Cancer Res. (2022) 82:3435–48. doi: 10.1158/0008-5472.CAN-22-0325, 35930804 PMC7613674

[13] Kim ES Velcheti V Mekhail T Yun C Shagan SM Hu S . Blood-based tumor mutational burden as a biomarker for atezolizumab in non-small cell lung cancer: the phase 2 B-F1RST trial. Nat Med. (2022) 28:939–45. doi: 10.1038/s41591-022-01754-x, 35422531 PMC9117143

[14] Kachroo S Shao C Desai K He J Jin F Sen S. Association of clinico-genomic characteristics with tumor mutational burden in small cell lung cancer patients. Future Oncol. (2021) 17:423–33. doi: 10.2217/fon-2020-0728, 33198513

[15] Shao MM Xu YP Zhang JJ Mao M Wang MC. Tumor mutational burden as a predictive biomarker for non-small cell lung cancer treated with immune checkpoint inhibitors of PD-1/PD-L1. Clin Transl Oncol. (2024) 26:1446–58. doi: 10.1007/s12094-023-03370-8, 38190035

[16] Raiber-Moreau EA Portella G Butler MG Clement O Konigshofer Y Hadfield J. Development and validation of blood tumor mutational burden reference standards. Genes Chromosomes Cancer. (2023) 62:121–30. doi: 10.1002/gcc.23100, 36326821 PMC10107199

[17] Gong JR Lee J Han Y Cho KH. DDX54 downregulation enhances anti-PD1 therapy in immune-desert lung tumors with high tumor mutational burden. Proc Natl Acad Sci USA. (2025) 122:e2412310122. doi: 10.1073/pnas.2412310122, 40172969 PMC12002276

[18] van den Heuvel GRM Kroeze LI Ligtenberg MJL Grünberg K Jansen EAM von Rhein D . Mutational signature analysis in non-small cell lung cancer patients with a high tumor mutational burden. Respir Res. (2021) 22:302. doi: 10.1186/s12931-021-01871-0, 34819052 PMC8611965

[19] Stabile LP Kumar V Gaither-Davis A Huang EH Vendetti FP Devadassan P . Syngeneic tobacco carcinogen-induced mouse lung adenocarcinoma model exhibits PD-L1 expression and high tumor mutational burden. JCI Insight. (2021) 6:e145307. doi: 10.1172/jci.insight.145307, 33351788 PMC7934870

[20] Ma K Huang F Wang Y Kang Y Wang Q Tang J . Relationship between tumor mutational burden, gene mutation status, and clinical characteristics in 340 cases of lung adenocarcinoma. Cancer Med. (2022) 11:4389–97. doi: 10.1002/cam4.4781, 35521981 PMC9678101

[21] Shi J Wang Z Zhang J Xu Y Xiao X Quan X . Genomic landscape and tumor mutational burden determination of circulating tumor DNA in over 5,000 Chinese patients with lung Cancer. Clin Cancer Res. (2021) 27:6184–96. doi: 10.1158/1078-0432.CCR-21-1537, 34446541

[22] Wehrle CJ Hong H Kamath S Schlegel A Fujiki M Hashimoto K . Tumor mutational burden from circulating tumor DNA predicts recurrence of hepatocellular carcinoma after resection: an emerging biomarker for surveillance. Ann Surg. (2024) 280:504–13. doi: 10.1097/SLA.0000000000006386, 38860385

[23] Caro-Vegas C Ramirez C Landis J Adimora AA Strickler H French AL . Molecular profiling of breast and lung cancer in women with HIV reveals high tumor mutational burden. AIDS. (2022) 36:567–71. doi: 10.1097/QAD.0000000000003144, 34873086 PMC8881359

[24] Ricciuti B Wang X Alessi JV Rizvi H Mahadevan NR Li YY . Association of High Tumor Mutation Burden in non-small cell lung cancers with increased immune infiltration and improved clinical outcomes of PD-L1 blockade across PD-L1 expression levels. JAMA Oncol. (2022) 8:1160–8. doi: 10.1001/jamaoncol.2022.1981. Erratum in: JAMA Oncol. 2022; 8(11):1702. doi:10.1001/jamaoncol.2022.5957, 35708671 PMC9204620

[25] Shi Y Lei Y Liu L Zhang S Wang W Zhao J . Integration of comprehensive genomic profiling, tumor mutational burden, and PD-L1 expression to identify novel biomarkers of immunotherapy in non-small cell lung cancer. Cancer Med. (2021) 10:2216–31. doi: 10.1002/cam4.3649, 33655698 PMC7982619

[26] Wu J Sun W Zhang Y Mao L Ding T Huang X . Impact of platinum-based chemotherapy on the tumor mutational burden and immune microenvironment in non-small cell lung cancer with postoperative recurrence. Clin Transl Oncol. (2024) 26:1738–47. doi: 10.1007/s12094-024-03397-5, 38421562

[27] Rosca OC Vele OE. Microsatellite instability, mismatch repair, and tumor mutation burden in lung Cancer. Surg Pathol Clin. (2024) 17:295–305. doi: 10.1016/j.path.2023.11.011, 38692812

[28] Strickler JH Hanks BA Khasraw M. Tumor mutational burden as a predictor of immunotherapy response: is more always better? Clin Cancer Res. (2021) 27:1236–41. doi: 10.1158/1078-0432.CCR-20-3054, 33199494 PMC9912042

[29] Li Y Ma Y Wu Z Zeng F Song B Zhang Y . Tumor mutational burden predicting the efficacy of immune checkpoint inhibitors in colorectal Cancer: a systematic review and Meta-analysis. Front Immunol. (2021) 12:751407. doi: 10.3389/fimmu.2021.751407, 34659255 PMC8511407

