# Supplementary material for: Correlation between tumor mutational burden and CT radiographic features in EGFR exon 19 deletion-mutated lung adenocarcinoma: a diagnostic accuracy study
Source: Front Med (Lausanne). 2026 May 29;13:1765136. doi: 10.3389/fmed.2026.1765136 (PMC13260179; doi:10.3389/fmed.2026.1765136)
Supplement: Supplementary file 1 [file Data_Sheet_1.docx]

# Supplementary Table S1: Stratified Analysis of the Combined Model's Diagnostic Performance for High TMB by Smoking Status

| Smoking Status | n (%) | Sensitivity (%) (95% CI) | Specificity (%) (95% CI) | PPV (%) (95% CI) | NPV (%) (95% CI) | Accuracy (%) (95% CI) | AUC (95% CI) |
| --- | --- | --- | --- | --- | --- | --- | --- |
| Never-Smokers | 78 (50) | 82.4 (65.5-93.2) | 78.7 (64.3-89.3) | 66.7 (50.5-80.4) | 89.8 (76.2-97.1) | 79.5 (68.8-87.8) | 0.82 (0.72-0.92) |
| Ever-Smokers | 78 (50) | 85.7 (69.7-95.2) | 75.0 (60.4-86.4) | 68.6 (52.3-82.1) | 89.2 (75.3-97.0) | 78.2 (67.4-86.8) | 0.81 (0.71-0.91) |
| Total Cohort | 156 (100) | 84.6 | 76.9 | 71 | 88.2 | 79.5 | 0.829 |
